# Anconeus and pronation: a palpatory and ultrasonographic study

**DOI:** 10.1007/s00276-024-03399-6

**Published:** 2024-07-23

**Authors:** Juan J. Canoso, Jorge Murillo-González, José Ramón Mérida-Velasco, Robert A. Kalish, Otto Olivas-Vergara, Cristina Gómez-Moreno, Eva García-Carpintero Blas, Gema Fuensalida-Novo, Esperanza Naredo

**Affiliations:** 1 Department of Medicine, ABC Medical Center, Mexico City. CDMX, Emeritus, Mexico; 2 Division of Rheumatology, Tufts University School of Medicine, Boston, MA USA; 3 Department of Anatomy and Embryology, Faculty of Medicine, Complutense University of Madrid, Madrid, 28040 Spain; 4 Department of Rheumatology and Bone and Joint Research Unit, Hospital Universitario Fundación Jiménez Díaz, IIS Fundación Jiménez Díaz, Madrid, Spain; 5 Department of Nursing, Hospital Universitario Fundación Jiménez Díaz, Madrid, Spain; 6 Universidad Autónoma de Madrid, Madrid, Spain

**Keywords:** Pronation, Anconeus, Pronator teres, Dynamic palpation, Dynamic ultrasonography, Dissection

## Abstract

**Purpose:**

Depending on its axis, pronation varies from the radius rotation around the steady ulna to the reciprocal adduction of the radius and abduction of the ulna. While there is no question that pronator teres is a central pronation agonist, anconeus’s role is not settled. The current investigation comparing palpation and ultrasonography in these two muscles during pronation along the axis capitulum-second digit evolved from a serendipitous finding in a clinical anatomy seminar.

**Methods:**

Single-hand palpation and two-transducer ultrasonography over anconeus and pronator teres were used on ten normal subjects to investigate their contraction during pronation around the capitulum-second digit axis. These studies were done independently and blind to the results of the other. The statistical analysis between palpation and ultrasonography was performed with Cohen’s kappa coefficient and the χ2 test.

**Results:**

On palpation, on resisted full pronation, anconeus contracted in 8/10 subjects and pronator teres in 10/10 subjects. Without resistance, the corresponding ratios were 5/10 and 9/10. On two-transducer ultrasonography, the comparable ratios were 7/10 and 10/10, and 3/10 and 10/10. A fair concordance (Cohen’s kappa = 0.21) between palpation and ultrasonography in detecting the simultaneous status of anconeus and pronator teres during resisted full pronation. Anatomic dissection illustrated the elements involved.

**Conclusions:**

Plain palpation confirmed by ultrasonography showed the simultaneous contraction of anconeus and pronator teres during resisted pronation in most of the studied subjects. The study suggests that palpation can be helpful in directly studying muscle activity during movement.

**Supplementary Information:**

The online version contains supplementary material available at 10.1007/s00276-024-03399-6.

## Introduction

Anconeus is a superficial triangular muscle located at the outer side and back of the elbow joint. Its tendon originates in the posterosuperior aspect of the lateral epicondyle of the humerus. It is close to the common extensor tendon; the fibers fan out and are attached to the olecranon and vicinity of the ulnar shaft [31]. With the elbow extended, anconeus is partially covered by extensor carpi radialis longus. However, the entire anconeus can be felt when the elbow is flexed [7, 13, 22, 34]. Triceps brachii and anconeus extend the elbow joint together. The contribution of anconeus to the total extension torque is only about 15% [2]. According to Duchenne [6], who made his observations based on direct faradic stimulation, “anconeus, whose action is quite powerful, contributes to all extension movements of the forearm. But this muscle also gives the ulna an outward movement, thanks to the obliquity of its fibers, which go from the epicondyle to the ulna, making its contribution very useful in pronation and supination movements”. Several authors [3, 7, 8, 12, 26] agreed with Duchenne´s observation. Also, pronation and supination may occur around the ulna, stressing the role of the interosseous membrane and rotation at the glenohumeral joint [29]. Contrasting with the cited authors, no participation of anconeus in pronation and only a minor contribution to elbow extension was shown in a detailed kinematic study in which anconeus action was blocked using lidocaine [21].

Finally, given its location and attachments, anconeus is widely considered a dynamic elbow stabilizer due to its compressive force [2, 16, 22, 27]. However, in a study of fourteen patients who had a supraclavicular block for wrist or hand surgery, the stabilizing function of triceps brachialis and particularly anconeus, although present, was minimal and considered below the threshold of clinical significance. Because anconeus has capsular insertions [4, 9, 14, 15, 22, 24], pulling the capsule back could prevent synovial catching during the extension of the humero-radial joint. This view is supported by the posterolateral plica syndrome that occurs from recurrent trauma during sports or heavy work. Anconeus’ various functions lead Bergin to describe it as “a multi-functional muscle at the elbow and forearm” [3].

The lateral part of the distal triceps and anconeus share a muscular and fascial connection [2–5]. Triceps brachii and anconeus are innervated by the radial nerve [18]. Anconeus receives a double innervation, a constant one through the nerve of the inner head of the triceps brachii and another inconstant from the posterior interosseous nerve (70.4%) [10]. Few anatomical variations regarding the morphology of anconeus have been described, such as the union, in varying degrees, with triceps brachii or with extensor carpi ulnaris. This muscle may also be absent or only represented by a fibrous band [18, 32, 33].

Pronator teres, is a superficial, rounded, obliquely arranged muscle with two heads. The humeral head that originates at the medial humeral epicondyle, and the medial intermuscular septum, and the antebrachial fascia. The ulnar head is smaller and originates at the coronoid process of the ulna. Both heads merge, forming a common tendon that inserts in the mid-shaft on the lateral surface of the radius [31]. By subtracting the pronation moment of pronator quadratus by the injection of lidocaine and with the elbow at 90-degree flexion, the overall pronator torque diminished by approximately 23%, suggesting that pronator teres is particularly important in maximal or resisted pronation [20]. Variations of pronator teres are uncommon and may involve its coronoid insertion; the muscle may be duplicated with a humeral and an ulnar bundle or may vary in the length of the radial insertion [18, 19, 25, 28, 30, 32]. While the median nerve passes between and innervates both heads [23, 26, 28], variations have been described in about 5% of specimens [30].

In the face of these sound, technologically complex, and well-executed studies, why did the authors dare publish this simple study of pronation based on palpation? The goal of our study, based on a serendipitous finding in a clinical anatomy seminar, was to investigate the synergy between the contraction of anconeus and pronator teres through palpation, a direct clinical tool, and ultrasonography to validate palpation.

## Materials and methods

### Ethics

The study was approved by the Ethics Committee of the Jiménez Díaz Foundation, project 28-06-2022, PIC117-22, and signed informed consents were obtained from all subjects.

The anatomical study was performed following the Declaration of Helsinki. The corpse belonged to the Center of Donation of Corpses, Complutense University of Madrid. All local and international ethical guidelines and laws regarding using human cadaveric donors in anatomical research were followed. Before death, all individuals gave written informed consent to use their donation for scientific purposes.

### Participants

Ten normal subjects, five females and five males, were studied by palpation and ultrasonography. The demographic features of the subjects are shown in Supplementary Information (Online Resource 1).

### Palpation

**Fig. 1:**
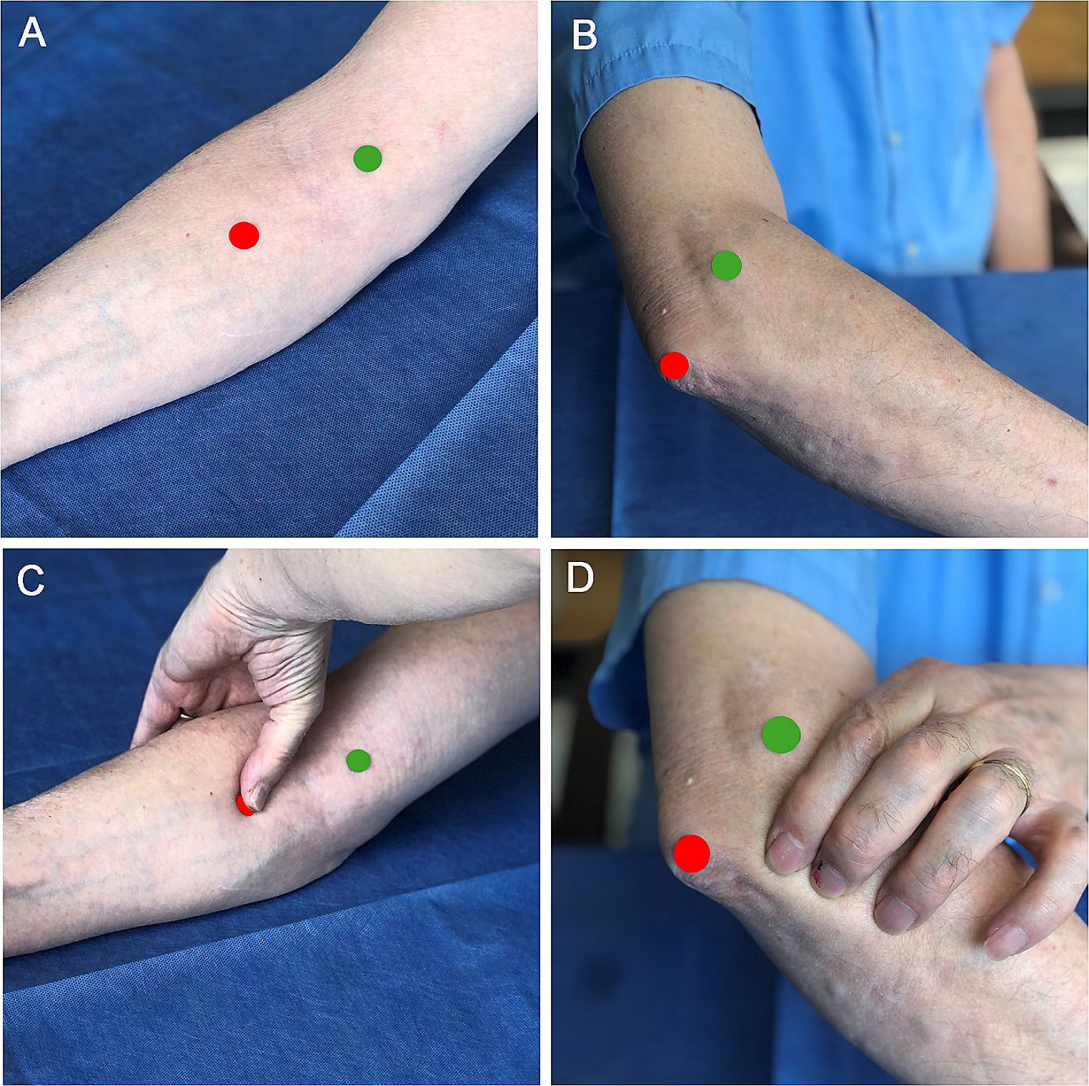
**A** Asking the subject to contract biceps brachii against resistance, the take-off of the bicipital aponeurosis from biceps brachii was marked with a green mark. The red mark 5 cm distal to this point is on pronator teres. **B** A green mark was placed on the lateral epicondyle and a red mark over the olecranon tip. The examiner´s hand, right or left, depending on whether the left or the right forearm was examined, embraced the forearm anteriorly with the thumb over pronator teres **C** and the index, middle, and ring fingers over anconeus **D**. Please note that the pictures shown were not obtained from a study subject but from a model

The participants’ dominant upper extremity was assessed while they were in the sitting position. Their arm was relaxed at their side, with the elbow flexed at a 90-degree angle and the palm facing upward near the edge of a table of standard height (74 cm). First, the take-off of the bicipital aponeurosis from biceps brachii was identified in the explored forearm while asking the subject to contract biceps brachii against resistance (Fig. 1A), and a mark was placed 5 cm distal to this point at the projection of pronator teres. Then, the mid-distance between the lateral epicondyle and the olecranon tip was marked at the projection of the proximal anconeus (Fig. 1B). After assuring that triceps was relaxed, the examiner´s hand, right or left, depending on whether the left or the right forearm was examined, embraced the forearm anteriorly with the web space of one hand, with the thumb over pronator teres (Fig. 1C) and the index, middle, and ring fingers over anconeus (Fig. 1D). Then, the subject was asked, in a continuous movement, to rotate the forearm along the capitulum-second digit axis from supination (thumb out) to neutral (thumb up) to full pronation (thumb in) and then exert an added pronation force by pressing the thumb into the table (resisted full pronation). Each subject performed this exercise three times to document the consistency of the findings. The palpatory findings at pronator teres and anconeus were graded as 0 (no contraction), and 1 (contraction) at each of the three positions and then during resisted pronation. Although contractions felt harder on resisted full pronation, quantification was impossible by palpation alone.

### Ultrasonography

**Fig. 2:**
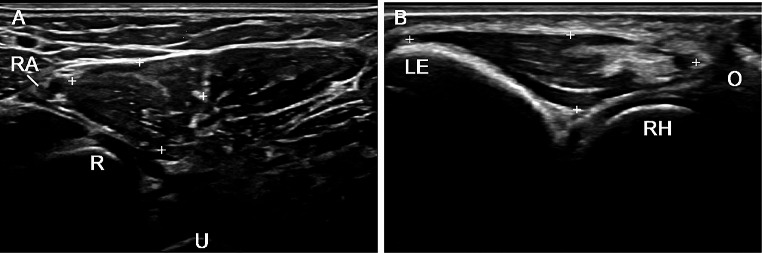
(**A**) Transverse scan of the pronator teres muscle (between crosses). (**B**) Longitudinal scan of the anconeus muscle (between crosses). LE, lateral epicondyle; R, radius; RH, radius head; RA, radial artery; U, ulna; O, olecranon

Two experts (EN, OO-V), with 27 and 5 years of experience in the musculoskeletal US, respectively, scanned the pronator teres and anconeus muscles with the transducer placed at the same anatomical locations used for palpation in both transverse and longitudinal planes. Muscle contraction was considered to occur when muscle thickness increased. The short axis of pronator teres was scanned in the anterior aspect of the forearm, oblique to the long axis of the forearm, and medial to the radial artery, sliding the transducer distally from the elbow joint. The long axis of pronator teres was visualized by sliding in the same area after rotating the probe 90º. The long axis of anconeus was scanned placing the transducer between the lateral epicondyle ant the olecranon and its short axis by rotating the probe 90º (Online Resource 2). A representative ultrasound image of each muscle is shown in Fig. 2. We used a real-time scanner (LOGIQ E9, GE Medical Systems Ultrasound and Primary Care Diagnostics, LLC, Wauwatosa, WI, USA) with a multifrequency linear transducer (ML 6–15 MHZ). B-mode settings were standardized for the study: B-mode frequency 15 MHz; B-mode gain 50 dB, and dynamic range 63 dB. After the identification of both muscles in their respective anatomical locations, the presence or absence of muscle contraction in supination, mid-pronation, full pronation, and resisted pronation was recorded in all subjects. As for palpation, ultrasound-detected muscle contraction was graded as 0 (no contraction) and 1 (contraction). Additionally, we evaluated the simultaneous contraction of both muscles during the pronation movement of the forearm, from supination to resisted full pronation, using a second US scanner (LOGIQ P10, GE Medical Systems Ultrasound and Primary Care Diagnostics, LLC, Wauwatosa, WI, USA equipped with a linear transducer ML 6–15 MHZ).

### Dissection

Pronator teres and anconeus dissection was performed bilaterally on a 76-year-old female embalmed corpse to show that the sites where the palpation and ultrasonography were carried out corresponded with the area where these muscles are located.

### Statistical analysis

The statistical analysis between palpation and ultrasonography was performed with Cohen’s kappa coefficient [17] in neutral, full, and resisted full pronation. The χ2 test was used to compare the contraction of anconeus and pronator teres as detected by palpation and ultrasonography in full and resisted full pronation.

## Results

### Palpation

Table 1 shows the palpatory findings at anconeus and pronator teres at each designated position during the arc of pronation. During the examination, it was observed that five out of ten subjects had anconeus contraction in full pronation. In comparison, eight out of ten subjects had the same contraction in resisted full pronation. Similarly, pronator teres contraction was noted in nine out of ten subjects in full pronation and all ten subjects during resisted full pronation. In one subject, pronator teres felt hard through the entire arc of pronation. In anconeus and pronator teres, the contractions felt harder when full pronation was resisted, while the distal end of the ulna was seen to rise slightly posteriorly; however, as mentioned, the degree of hardness and the rise of the ulna could not be measured.

**Table 1:** Contraction of anconeus and pronator teres on simultaneous palpation

Subject number	1	2	3	4	5	6	7	8	9	10
**Anconeus**										
Supination	0	0	0	0	0	0	0	0	0	0
Neutral	0	0	0	0	0	1	0	1	0	1
Full pronation	0	0	0	0	1	1	1	1	0	1
Resisted full pronation	1	1	0	0	1	1	1	1	1	1
**Promator teres**										
Supination	0	0	0	1	0	0	0	0	0	0
Neutral	1	0	0	0	0	1	1	1	1	1
Full pronation	1	0	1	1	1	1	1	1	1	1
Resisted full pronation	1	1	1	1	1	1	1	1	1	1

0 = no contraction, 1 = contraction

### Ultrasonography

The US readily identified pronator teres and anconeus at their anatomical locations in all forearm positions (Fig. 2). In Table 2, as determined by simultaneous two-transducer ultrasonography, three out of ten subjects showed anconeus contraction during full pronation, and seven out of ten on resisted full pronation. In contrast, all ten subjects demonstrated pronator teres contraction during full and resisted full pronation. Two videos (Online Resources 3, 4 and 5) show the simultaneous contraction of anconeus and pronator teres during full pronation and how their thickness increased when an opposing external force was applied.

**Table 2:** Contraction of anconeus and pronator teres on simultaneous (two-transducer) ultrasonography

Subject number	1	2	3	4	5	6	7	8	9	10	
**Anconeus**											
Supination	0	0	0	0	0	0	0	0	0	0	
Mid pronation	0	0	1	0	1	0	0	0	0	0	
Full pronation	0	0	1	0	1	0	1	0	0	0	
Resisted full pronation	1	0	1	0	1	1	1	1	0	1	
**Pronator teres**											
Supination	0	0	0	0	0	0	0	0	0	0	
Mid pronation	1	1	1	1	1	1	1	1	0	1	
Full pronation	1	1	1	1	1	1	1	1	1	1	
Resisted full pronation	1	1	1	1	1	1	1	1	1	1	

0 = no contraction, 1 = contraction

### Dissection

**Fig. 3:**
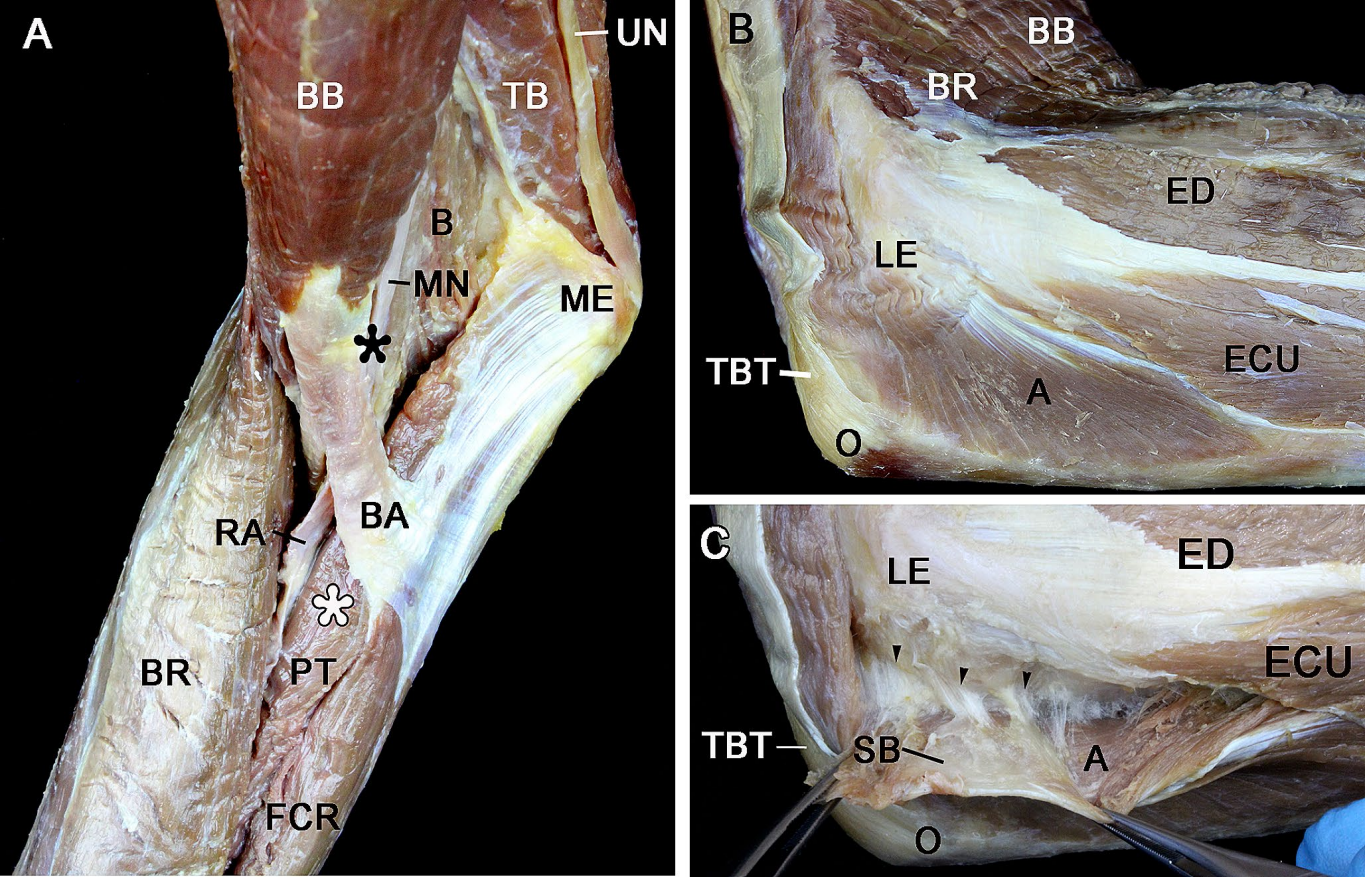
**A** and **B** Right upper limb dissection demonstrating pronator teres (PT) and anconeus (**A**). **C** The anconeus (**A**) has been removed from the posterior aspect of the lateral epicondyle (LE) to show its adherences with the fibrous capsule of the elbow joint (arrowheads). Black asterisk, proximal border of the bicipital aponeurosis as it takes off from biceps brachii; White asterisk, 5 cm distal to the black asterisk overlapping pronator teres; B, brachialis; BA, bicipital aponeurosis; BB, biceps brachii; BR, brachioradialis; ECU, extensor carpi ulnaris; ED, extensor digitorum; FCR, flexor carpi radialis; MN, median nerve; ME, medial epicondyle; O, olecranon; RA, radial artery; SB, serous bursa; TB, triceps brachii; TBT, triceps brachii tendón; UN, ulnar nerve

The anterior regions of the elbow and forearm were dissected, exposing pronator teres insertion in the anterior surface of the medial epicondyle via the common tendon of origin of the flexor muscles. Pronator teres limited the cubital fossa medially and passed obliquely across the forearm to reach its insertion in the middle third of the radius. The bicipital aponeurosis, used as a reference point to identify pronator teres by palpation, was also seen (Fig. 3A). The posterior regions of the elbow and forearm were dissected, exposing anconeus. The reference points for palpation, the olecranon, and the lateral epicondyle were identified (Fig. 3B). Anconeus was sharply elevated from its origin in the posterior aspect of the lateral epicondyle to identify adherences to the fibrous capsule of the elbow joint (Fig. 3C).

### Statistical analysis

A fair concordance (Cohen’s kappa = 0.21) was noted between palpation and ultrasonography regarding the status of anconeus and pronator teres during resisted full pronation. Concordance was slight for both methods during full pronation (Cohen´s kappa = 0.12). During the neutral position, Cohen’s kappa was − 0.176. Compared to full pronation, anconeus, and pronator teres, contractions were more frequently detected during resisted full pronation either by palpation or by ultrasonography, which was particularly evident for anconeus. By palpation, detection was 100% vs. 90% for pronator teres and 80% vs. 50% for anconeus. By ultrasonography, 100% vs. 100% for pronator teres and 70% vs. 30% for the anconeus. However, none of these comparisons reached statistical significance with the χ2 test due to the low number of events evaluated.

## Discussion

This study was triggered by the serendipitous finding of the current contraction of anconeus and pronator teres in a clinical anatomy seminar consisting of the cross-examination of instructors and participants [11]. To explore these early findings, we compared the simultaneous palpation of anconeus and pronator teres with two-transducer ultrasonography, which depicted both muscles at the explored sites. Our findings confirmed the initial hypothesis of a concurrent contraction of pronator teres and anconeus in most participants by palpation and ultrasonography. The contraction of both muscles in full and especially resisted full pronation is clearly shown in Videos S1 and S2 (Online Resources 3, 4 and 5). In addition, abduction of the ulna was verified by inspection by watching a slight, unquantifiable posterolateral movement of the distal ulna in resisted full pronation.

Our clinical and ultrasonographic study benefitted from having two experienced anatomists (JMG and JRMV) on site. The ultrasound component added certainty to the palpatory findings, and the anatomic dissections gave a clear view of the explored structures, including the relationship of anconeus with the elbow’s joint capsule.

Pronation has fascinated researchers for over one and a half centuries, not only because of the impact of the pronation axis but also of the muscles involved. Classically, pronation is achieved by the concerted action of pronator teres in the proximal forearm and pronator quadratus in the distal forearm with the addition of brachioradialis if the forearm is already in supination. Muscles that may contribute to pronation torque include anconeus, mainly when the axis of pronation is around the capitellum-second finger axis, pronator teres, pronator quadratus, brachioradialis, extensor carpi radialis brevis, deltoid, and potentially finger flexors and wrist flexors. The complexity of pronation has long been recognized [26, 34]. An exciting finding in pronator teres was that the activity of the deep head was present in both pronation and supination, supporting the view that this head is a dynamic stabilizer of the distal radioulnar joint. These investigators commented that pronation may imply a “complex and redundant system that is not readily analyzed based on EMG” [5].

It has been stated that only one nerve is necessary for pronation, the median for pronator teres and pronator quadratus. In contrast, supination requires two nerves, the radial for supinator and the musculocutaneous for biceps brachialis [12]. If our observations and others’ are confirmed, the radial nerve also contributes to pronation.

There are several limitations to our study. One is the small number of observations that did not allow for the assessment of interrater reliability or the effect of age, gender, physical activity, etc. However, both the clinical and ultrasonography assessors had extensive and intensive experience in the field, which may have reduced variability. Another limitation is the qualitative nature of the observations, which may have been a benefit since palpation represents a direct approach to the question of whether anconeus contracts or not during pronation. An additional limitation is the rudimentary way in which muscle contraction was quantified. A stiffness measurement tool [1] could have helped, but it was unfortunately unavailable to the investigator. On the other hand, palpation provides a direct haptic observation without an interposed technology that could have limitations, such as adapting a rigid frame to a complex anatomy subject to subtle changes during motion. A question raised by one of our anatomists was whether anconeus contracted or was just lifted by a clamping effect of the adjacent bones during pronation. However, on palpation, the thickened tissue felt like a muscle, and it was easy to learn to tense the forearm muscles without motion, with finding at anconeus that were identical to the contraction noted during full and resisted full pronation.

A concern regarding palpation as a tool for anatomic investigation is the poor knowledge of clinical anatomy in rheumatology and trainees in related specialties our group assessed along the Americas, from the US to Uruguay, Argentina, and Chile, years before the COVID-19 pandemic [23]. We believe that, provided sufficient anatomical knowledge, palpation, which can encircle a large portion of a limb´s circumference or be conducted bimanually, in association with ultrasonography, may lead to a better understanding of functional anatomy. For example, based on our findings, we hypothesize that studies of nerve branching patterns [35] or the region of highest spindle abundance [36], when planning the treatment of forearm spasticity, which includes pronation, should include anconeus.

## Conclusions

We studied anconeus and pronator teres by palpation and two-transducer ultrasonography during pronation around the capitulum-second digit axis of forearm rotation. Both muscles contracted in late pronation, sooner in pronator teres, and more clearly on resisted full pronation.

## Electronic supplementary material

Below is the link to the electronic supplementary material.

Supplementary Material 1

Supplementary Material 2

Supplementary Material 3

Supplementary Material 4

Supplementary Material 5

## Data Availability

The data supporting this study?s findings are available from the corresponding author upon reasonable request.
