# Supplementary material for: Anconeus and pronation: a palpatory and ultrasonographic study
Source: Surg Radiol Anat. 2024 Jul 23;46(9):1447–54. doi: 10.1007/s00276-024-03399-6 (PMC11424725; doi:10.1007/s00276-024-03399-6)
Supplement: Supplementary file 1 — Supplementary Material 1 [file 276_2024_3399_MOESM1_ESM.docx]

**Table S1** Demographic and clinical data in ten healthy subjects

| **Subject number** | | | | | | | | | | |
| --- | --- | --- | --- | --- | --- | --- | --- | --- | --- | --- |
|  | 1 | 2 | 3 | 4 | 5 | 6 | 7 | 8 | 9 | 10 |
| Sex | F | M | F | M | M | M | M | F | F | F |
| Age | 40 | 62 | 40 | 58 | 38 | 58 | 53 | 53 | 52 | 58 |
| Height | 167 | 180 | 173 | 178 | 170 | 178 | 174 | 168 | 156 | 152 |
| Weight | 65 | 84 | 70 | 98 | 72 | 76 | 83 | 56 | 68 | 52 |
| BMI | 23.3 | 25.9 | 23.4 | 30.9 | 24.9 | 24 | 27.4 | 19.8 | 27.9 | 22.5 |
| Dominant hand | R | R | R | L | L | R | R | R | R | R |
