# Supplementary figures and images for: Anconeus and pronation: a palpatory and ultrasonographic study
Source: Surg Radiol Anat. 2024 Jul 23;46(9):1447–54. doi: 10.1007/s00276-024-03399-6 (PMC11424725; doi:10.1007/s00276-024-03399-6)

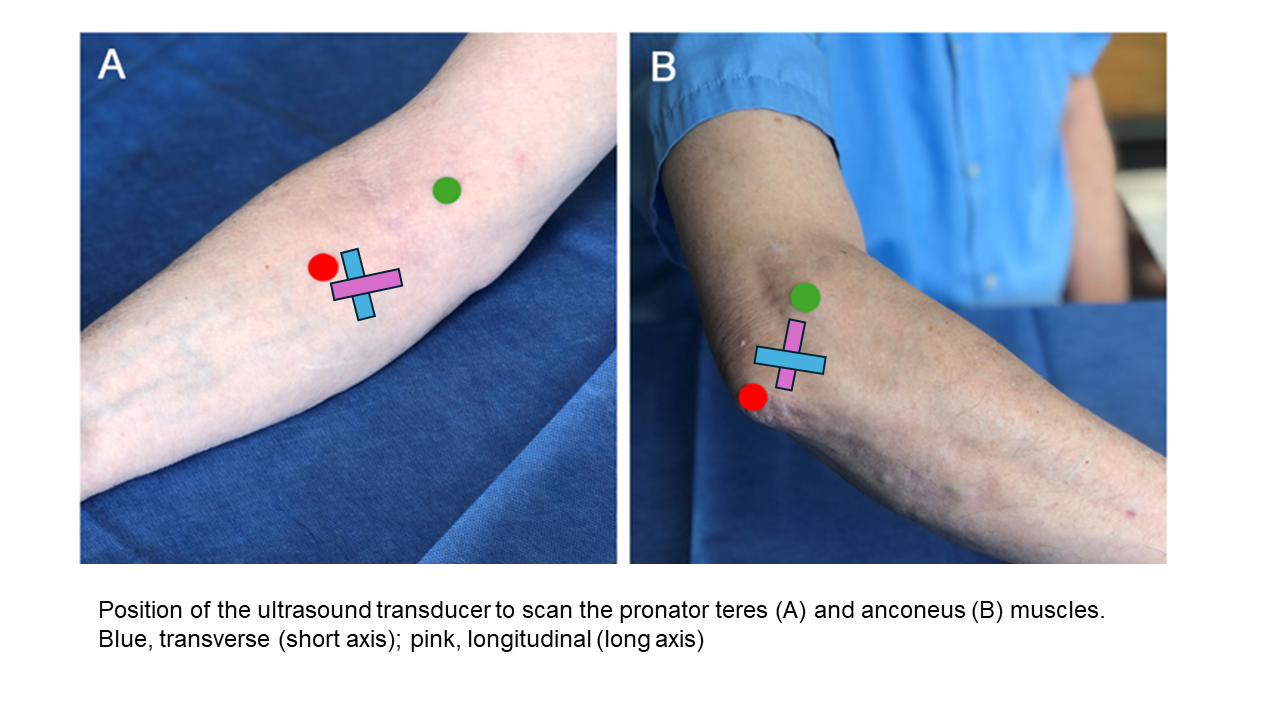

Supplement: Supplementary file 2 — Supplementary Material 2 [file 276_2024_3399_MOESM2_ESM.png]

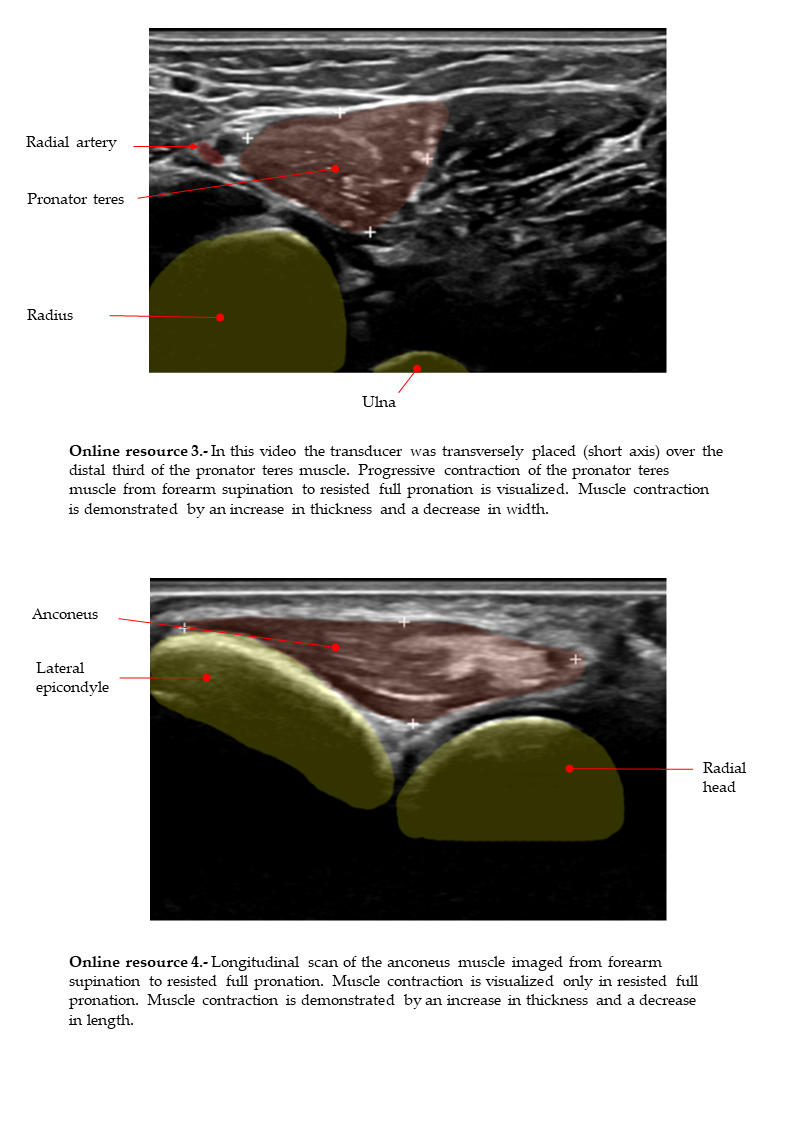

Supplement: Supplementary file 5 — Supplementary Material 5 [file 276_2024_3399_MOESM5_ESM.png]
